# Functional genomics study of *Pseudomonas putida* to determine traits associated with avoidance of a myxobacterial predator

**DOI:** 10.1038/s41598-021-96046-8

**Published:** 2021-08-12

**Authors:** Shukria Akbar, D. Cole Stevens

**Affiliations:** Department of BioMolecular Sciences, University of Mississippi, University, MS USA

**Keywords:** Applied microbiology, Environmental microbiology, Soil microbiology

## Abstract

Predation contributes to the structure and diversity of microbial communities. Predatory myxobacteria are ubiquitous to a variety of microbial habitats and capably consume a broad diversity of microbial prey. Predator–prey experiments utilizing myxobacteria have provided details into predatory mechanisms and features that facilitate consumption of prey. However, prey resistance to myxobacterial predation remains underexplored, and prey resistances have been observed exclusively from predator–prey experiments that included the model myxobacterium *Myxococcus xanthus*. Utilizing a predator–prey pairing that instead included the myxobacterium, *Cystobacter ferrugineus,* with *Pseudomonas putida* as prey, we observed surviving phenotypes capable of eluding predation. Comparative transcriptomics between *P. putida* unexposed to *C. ferrugineus* and the survivor phenotype suggested that increased expression of efflux pumps, genes associated with mucoid conversion, and various membrane features contribute to predator avoidance. Unique features observed from the survivor phenotype when compared to the parent *P. putida* include small colony variation, efflux-mediated antibiotic resistance, phenazine-1-carboxylic acid production, and increased mucoid conversion. These results demonstrate the utility of myxobacterial predator–prey models and provide insight into prey resistances in response to predatory stress that might contribute to the phenotypic diversity and structure of bacterial communities.

## Introduction

Abundant within soils and marine environments, predatory myxobacteria contribute to nutrient cycling within the microbial food web^1–4^. Myxobacteria are prolific producers of antimicrobial specialized metabolites and display a cooperative, swarming predation strategy that can be readily reproduced and monitored within laboratory settings making them uniquely appropriate for assessment of predator–prey interactions^1,3–5^. Broadly considered generalist predators, myxobacteria capably predate a range of prey including both Gram-negative and Gram-positive bacteria as well as fungi^5,6^. Constitutive production of specialized metabolites and lytic proteins are often associated with this predatory range, and a variety of metabolites and enzymatic features have been reported to benefit predation^7–10^.

However, relatively few examples of prey resistance to myxobacterial predation have been reported^11^. Compared to features associated with bacterial strategies to avoid protozoan predators, prey avoidance of predatory myxobacteria remains underexplored^12–16^. Examples of prey responses correlated with resistance to myxobacterial predation include *Escherichia coli* biofilm formation^17^, *Bacillus subtilis* sporulation and production of bacillaene^18,19^, *Bacillus licheniformus* glycosylation of the predation-associated metabolite myxovirescin A^20^, galactoglucan exopolysaccharide production and increased melanin production by *Sinorhizobium meliloti*^21,22^, and formaldehyde secretion by *Pseudomonas aeruginosa*^11^. All of these features were discovered from predator–prey experiments utilizing the model myxobacterium *M. xanthus*.

Considering these diverse mechanisms opted by different prey organisms, we suspect the development of predator–prey pairings including other myxobacterial prey might provide additional insight into prey resistance to myxobacterial predation. For our predator–prey experiments, the soil dwelling myxobacterium *Cystobacter ferrugineus* strain Cbfe23 was included due to a favorable growth profile and capability to quickly consume *Pseudomonas putida* during standard predation assays^23–25^. Also found within soils, root colonizing *P. putida* was chosen as prey due to an established ability to resist protozoan grazers^14^. Initial predator–prey experiments provided a *P. putida* phenotype capable of avoiding *C. ferrugineus* predation. Herein we report the generation and predator avoidance of a *P. putida* phenotype resistant to myxobacterial predation using standard predator–prey experiments, differential gene expression data comparing the survivor phenotype with predator-unexposed *P. putida*, and traits observed to potentially contribute to predator avoidance.

## Results

### Selection of P. putida phenotypes that avoid predation

**Figure 1:**
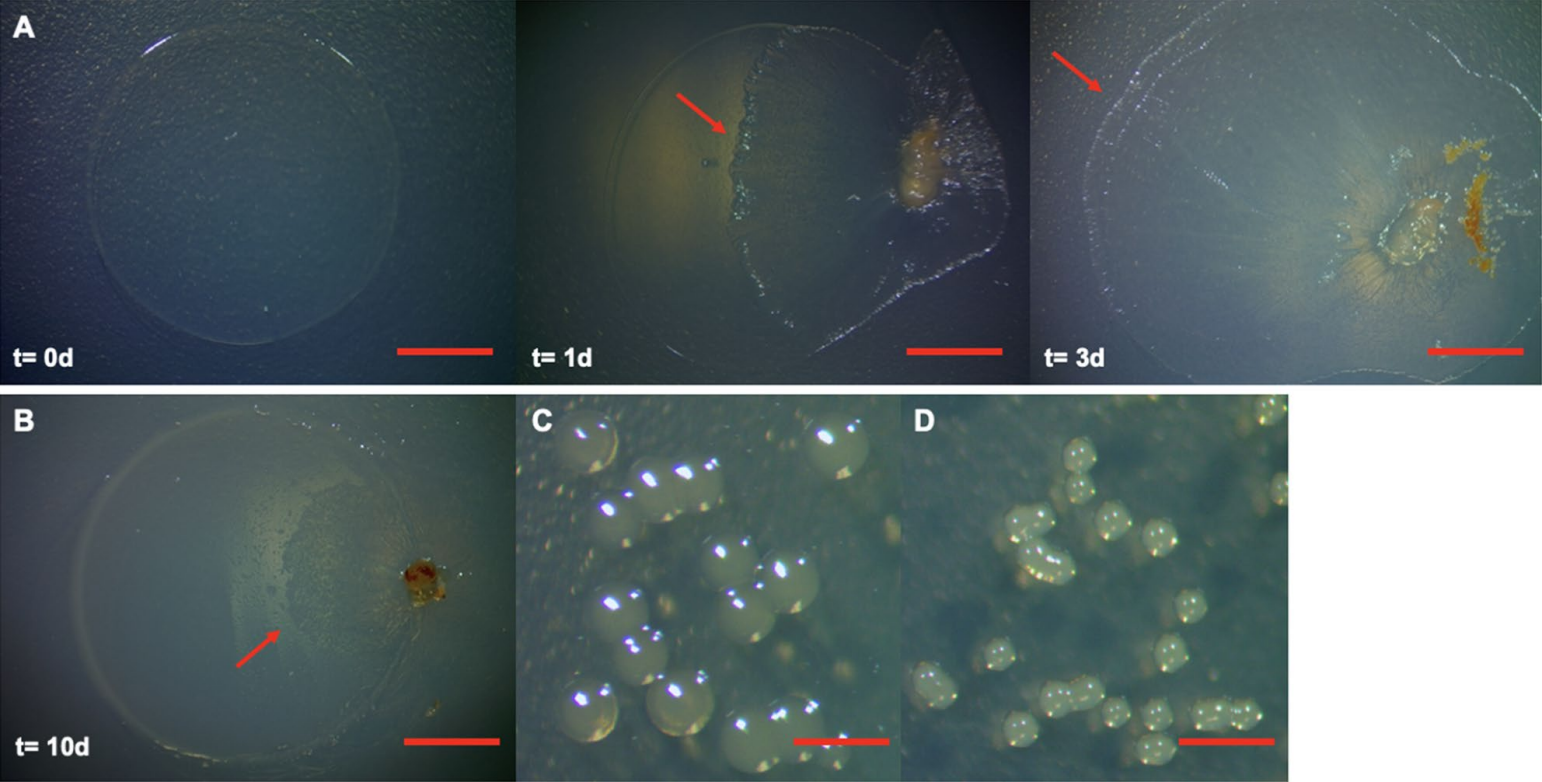
Predation assays resulting in initial observation of survivor *P. putida*. (**A**) Three day sequence of a *C. ferrugineus*-*P. putida* predation assay depicting complete swarming of spotted prey lawns with no visible parent *P. putida* biomass; red arrows indicate *C. ferrugineus* swarm edge. Day 0 panel is only *P. putida* biomass prior to introduction of *C. ferrugineus*. (**B**) Day 10 of *C. ferrugineus*-*P. putida* predation assay depicting incomplete swarming of prey lawns; red arrow indicates *C. ferrugineus* swarm edge. Comparison of colony sizes of (**C**) parent *P. putida* colonies and (**D**) survivor *P. putida* colonies depicting small colony variation. All scale bars depict 1 mm.

Predatory stress from the prokaryotic and eukaryotic predators selects for predation avoidance traits in the prey bacteria^12–22^. Previously, *P. putida* has exhibited resistance to the predation by protozoan grazers^14^. To identify any such predation avoidance of *P. putida* when interacting with a bacterial predator, we chose to utilize a culture-based predation assay on solid agar media and investigated the predatory interaction between a predatory myxobacterium *C. ferrugineus* and *P. putida* prey. Utilizing predation assays where an inoculum of *C. ferrugineus* was introduced to the edge of an established spot of *P. putida*^24^, we considered swarming overtaking the *P. putida* spot with no visible prey biomass remaining to be an endpoint of predation. Typically, *C. ferrugineus* swarmed over *P. putida* with no visible prey biomass after 3 days of co-cultivation on nutrient-free WAT agar (Fig. 1A). However, one spot of *P. putida* from the initial ten assays provided minimally consumed and readily observable biomass of *P. putida* remaining on the assay plate even after 14 days of co-culturing (Fig. 1B). To access the viability of this remaining *P. putida* biomass, we cultured it on an LB agar plate. Interestingly, the resulting colonies were smaller compared to the *P. putida* parent strain that we utilized in this study (Fig. 1C,D) and appeared after 36 h compared to the 18 h incubation time of parent *P. putida*.

Attempting to reproduce this *P. putida* survivor phenotype, we prepared 90 additional predation assays with all prey spots originating from individual colonies of *P. putida*. Among these, 50 assays were performed on nutrient-free WAT agar and 40 assays on nutrient-rich VY/2 agar plates. From these assays, we again found minimally consumed and readily observable biomass of *P. putida* twice each from the nutrient-free and nutrient-rich media which suggests a survivor *P. putida* frequency of 4–5% on nutrient-free and nutrient-rich medias respectively. Cells from the remaining biomass of *P. putida* prey were also cultivated on LB agar, and again the colonies from each individually collected prey phenotype were noticeably smaller than parent *P. putida*. Notably, microcolonies or small colony variants are previously observed for *Pseudomonas* sp. CM10, *Pseudomonas aeruginosa*, and *Serratia marcescens* and protected them from their protozoan grazers^26–28^. Comparative growth curves revealed a 1.8 h doubling time for parent *P. putida* and a 2.9 h doubling time for the small colony variant phenotype as well as a lower final biomass for survivor *P. putida* (Supplemental Fig. 2). For clarity, we will refer to this small colony variant as survivor *P. putida* and the predation susceptible *P. putida* type strain as parent *P. putida*. Stocks of colonies obtained after culturing each replicate of *P. putida* survivor observed from predation assays were stored at − 80 °C as an individual aliquot and utilized for further analysis.

**Figure 2:**
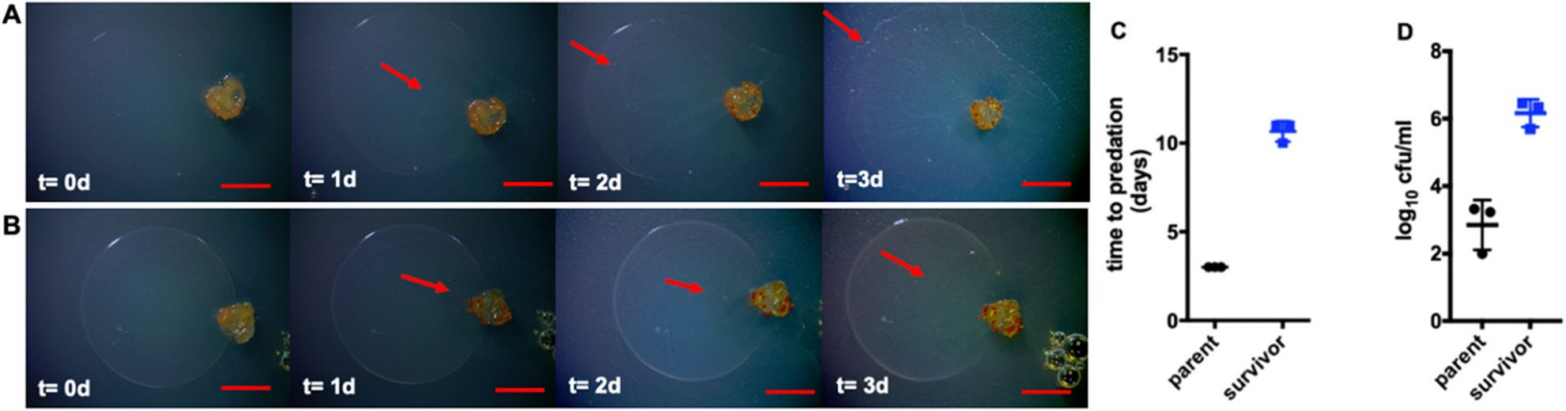
Predation assays depicting predator avoidance of survivor *P. putida* on nutrient-free media. (**A**) Predation assay depicting complete swarming of *C. ferrugineus* on spotted prey lawns with no visible parent *P. putida* biomass after 3 days (t = d3) red arrows indicate *C. ferrugineus* swarm edge. (**B**) Predation assay depicting predator avoidance observed from survivor *P. putida* lawns and visible biomass remaining with minimal *C. ferrugineus* swarming after 3 days (t = d3); red arrows indicate *C. ferrugineus* swarm edge. (**C**) Time to predation data for parent *P. putida* and survivor *P. putida* (n = 3; *p* ≤ 0.002) with (**D**) end point CFU data (n = 3; *p* ≤ 0.006) to determine differences in cell viabilities post-swarming. Unpaired t-test with Welch’s correction used for statistical analyses included in (**C**,**D**); all scale bars depict 1 mm.

### Survivor P. putida eludes subsequent predation

To confirm the predator avoidance associated with survivor *P. putida* in our study, we performed subsequent predation assays. The predation assay was performed as described previously, with predator cells directly introduced to the edge of the established prey spot on a nutrient-free WAT agar^24^. We considered swarming overtaking the *P. putida* spot with no visible prey biomass remaining to be an endpoint of predation. Typically, *C. ferrugineus* swarmed parent *P. putida* with no visible biomass left after 3 days of co-cultivation on nutrient-free WAT agar (Fig. 2A,C). However, *C. ferrugineus* took relatively longer to swarm survivor *P. putida* biomass (Fig. 2B,C).

To assess predation efficiency post-swarming of prey biomass, we performed colony forming unit (CFU) assays to determine remaining viable prey cells. Exponentially fewer viable colonies of parent *P. putida* were observed from endpoint CFU assays (3–4 days for parent *P. putida*; 10–14 days for survivor *P. putida*) (Fig. 2C,D). This suggests that even though *C. ferrugineus* slowly swarmed survivor *P. putida* over 14 days, the predation efficiency also remained lower. Also apparent from CFU assays, colonies of survivor *P. putida* remained consistently smaller when compared to parent *P. putida*.

**Figure 3:**
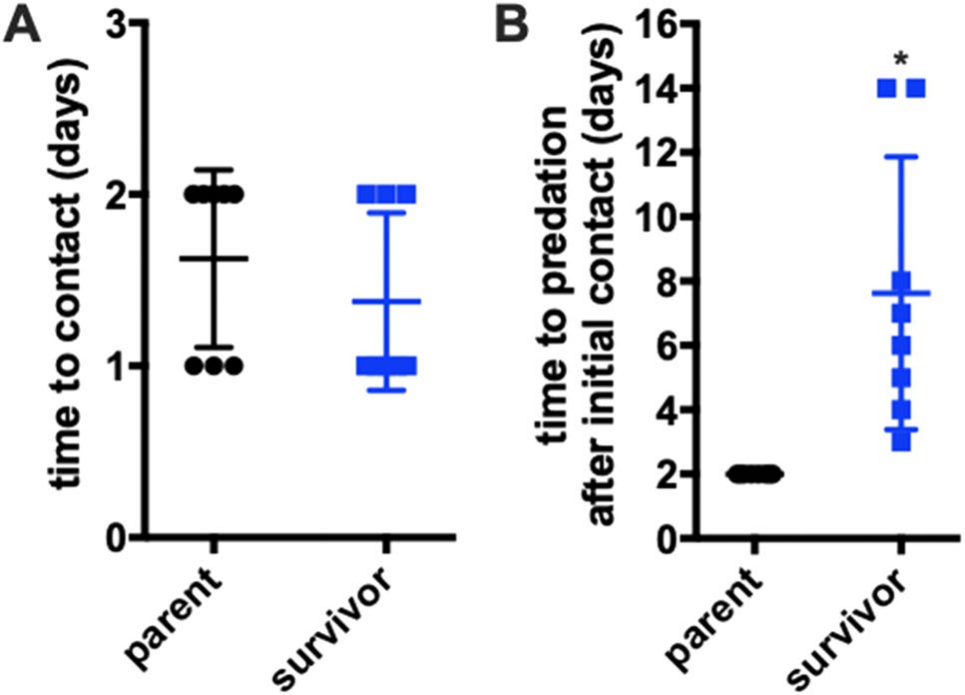
Predatory avoidance trait of survivor *P. putida* is contact dependent. Modified distant spot predation assay data with prey introduced 2 cm from *C. ferrugineus* depicting (**A**) no statistical differences in time to predator–prey contact (n = 8) and (**B**) significant differences in time to predation after contact (predation considered complete swarming of prey biomass) (n = 8; *p* ≤ 0.01). Statistical significance calculated using an unpaired t test with Welch’s correction.

Noticing less efficient predation of survivor *P. putida*, we investigated the time that *C. ferrugineus* would take to approach survivor *P. putida* compared to parent *P. putida*. For this a slightly modified distant spot predation assay was performed with *C. ferrugineus* introduced at a distance of 5–7 mm from an established spot of *P. putida*. For distant spot predation assays, time to predator–prey contact and time to complete swarming of prey biomass were recorded. *C. ferrugineus* showed directed movement towards survivor *P. putida* similar to parent *P. putida* and time to predator–prey contact was between 1 and 2 days for both phenotypes (Fig. 3A). This result precludes the contribution of a diffusible metabolite contributing to the predator avoidance trait of survivor *P. putida.* However, after initial contact with prey, *C. ferrugineus* swarming was again slower over survivor *P. putida* compared to parent *P. putida* (Fig. 3B) suggesting that direct contact is required for the predator avoidance trait.

### Nutrient availability does not diminish predator avoidance

**Figure 4:**
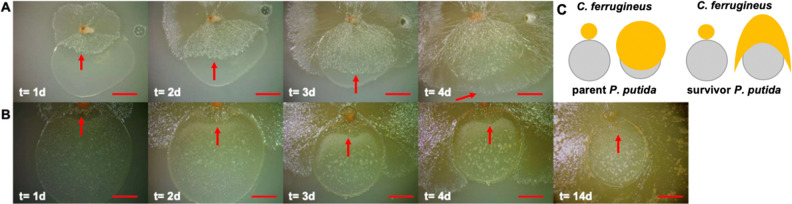
Survivor *P. putida* maintains predator avoidance trait on nutrient rich media. (**A**) 4 day sequence of *C. ferrugineus* predation of parent *P. putida* on nutrient-rich VY/2 media depicting complete swarming of prey lawn (t = d4); red arrows indicate *C. ferrugineus* swarm edge progression into parent *P. putida* lawn. (**B**) 4 day sequence depicting predator avoidance observed from survivor *P. putida* lawn on nutrient-rich VY/2 media including an additional image from day 14 to demonstrate length of avoidance; red arrows indicate *C. ferrugineus* swarm edge progression into survivor *P. putida* lawn. (**C**) Cartoon depicting difference in *C. ferrugineus* swarming patterns between parent *P. putida* (**A**) and survivor *P. putida* (**B**). All scale bars depict 1 mm.

Since we originally observed selection of survivor *P. putida* on VY/2 nutrient-rich media, we also performed predation assays on VY/2 media to determine any change in predator avoidance that might be attributable to nutrient availability. When compared to parent *P. putida*, *C. ferrugineus* was mostly unable to swarm survivor *P. putida* during predation assays on nutrient-rich media with little to no prey biomass degradation visible (Fig. 4). Additionally, instead of evenly distributed frontal swarming of parent *P. putida*, *C. ferrugineus* swarmed along the perimeter of survivor *P. putida* (Fig. 4). Although we observed a similar difference in swarming during our assays on nutrient-free media (Fig. 2), these assays provided a more apparent contrast. These results and the observed differences in swarming from *C. ferrugineus* during predator–prey interactions parallel previous observation of mucoid *S. meliloti* phenotypes that avoid *M. xanthus* predation^22^. Overall these data demonstrate that survivor *P. putida* maintains the predator avoidance trait on nutrient-rich media.

### Increased pyoverdine production, mucoid conversion, and antibiotic resistance observed from survivor P. putida

**Figure 5:**
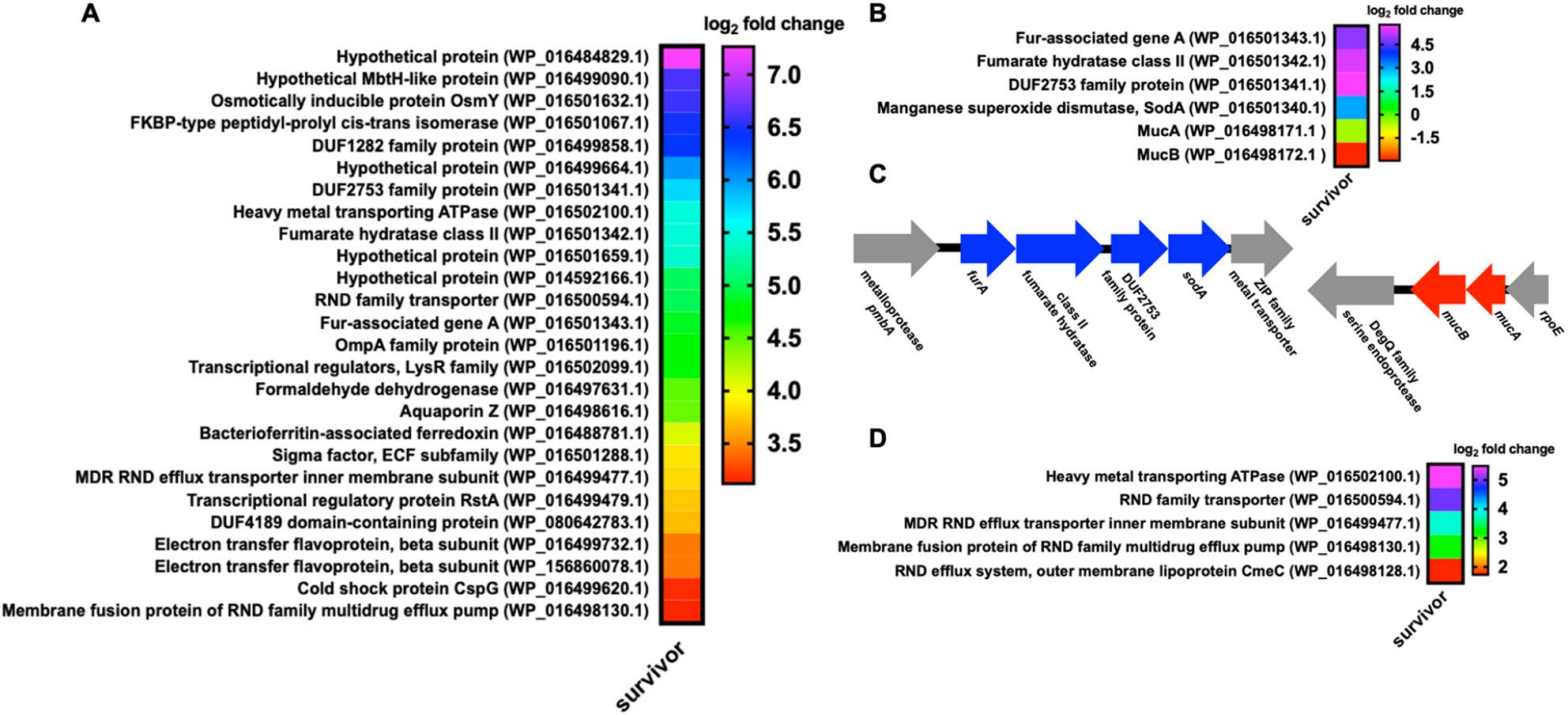
Upregulated features from survivor *P. putida* include multiple features associated with the AglU operon and efflux components. (**A**) Most abundantly over-expressed genes from survivor *P. putida* when compared to parent *P. putida*. (**B**) Up-regulated features associated with alginate production and the associated genomic context for each (**C**). (**D**) Up-regulated transport proteins including those associated with antibiotic efflux. All data depicted as an averages from 3 biological replicates comparing survivor *P. putida* with parent *P. putida* (*p* ≤ 0.05).

Utilizing RNA sequencing, we conducted comparative transcriptomic experiments to identify differentially regulated genes comparing parent *P. putida* and survivor *P. putida*. For this analysis, we employed 3 samples collected independently out of 5 initially observed and stored survivor *P. putida* isolates. This comparative analysis determined that 1,148 genes were down-regulated ≥ fourfold and 153 genes were up-regulated ≥ fourfold across survivor *P. putida* replicates when compared to parent *P. putida* replicates (n = 3; *p* ≤ 0.05). Considering up-regulated genes from survivor *P. putida* with the highest fold change values, apparent differences in overexpressed features specific to the survivor phenotype included genes involved in siderophore production, mucoid conversion, and antibiotic efflux (Fig. 5).

**Figure 6:**
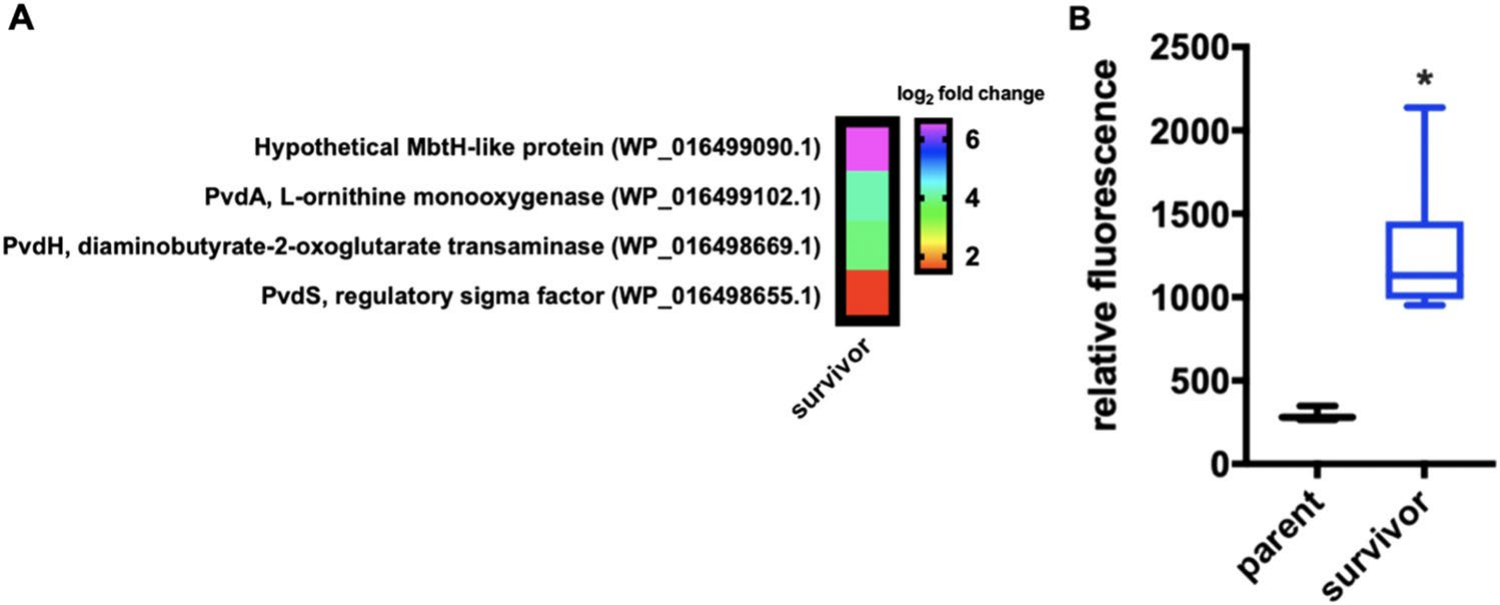
Pyoverdine features up-regulated in survivor *P. putida*. (**A**) Features associated with pyoverdine biosynthesis up-regulated in the survivor phenotype. Data depicted as an averages from 3 biological replicates comparing survivor *P. putida* with parent *P. putida* (*p* ≤ 0.05). (**B**) Relative fluorescence of extracellular pyoverdine depicting increased pyoverdine production from the survivor phenotype (n = 12; *p* ≤ 0.0001). Relative units determined from average of control values with.

Features associated with the biosynthesis of pyoverdine, a siderophore associated with iron acquisition, were significantly up-regulated in survivor *P. putida*. These included PvdA an L-ornithine monooxygenase (WP_016499102.1) and PvdH a diaminobutyrate-2-oxoketoglutarate transaminase (WP_016498669.1), both involved in pyoverdine precursor biosynthesis, a regulatory transcription factor PvdS (WP_016498655.1), and a proximal MbtH-like protein (WP_016499090.1) (Fig. 6A)^29–32^. To determine if increased expression of these features resulted in increased production of pyoverdine, fluorescence of extracellular pyoverdine was quantified from clarified media of parent *P. putida* and survivor *P. putida* using methodology established by Imperi *et al*^33,34^. Indicative of increased pyoverdine secretion, extracellular fractions from survivor *P. putida* exhibited a three–fivefold increase in fluorescence when compared to extracellular fractions from parent *P. putida* (Fig. 6B). Although genome data from parent *P. putida* includes a pyoverdine biosynthetic gene cluster, no structurally elucidated pyoverdines have been characterized from the *P. putida* type strain, and these results merely suggest the presence of fluorescent pyoverdine-like metabolites potentially produced by the strain. Regardless, these results corroborate overexpression of pyoverdine biosynthetic pathway components observed in our transcriptomic analysis and suggest that siderophore pyoverdine production may contribute to the predator avoidance trait of survivor *P. putida*.

Also associated with iron acquisition and iron limiting conditions, a TonB-dependent hemin, ferrichrome receptor (WP_016501290.1), a ferric iron ABC transporter (WP_016501876.1), a bacterioferritin (WP_016501855.1), and a bacterioferritin-associated ferredoxin (WP_016488781.1) were upregulated in survivor *P. putida*. The TonB-dependent hemin, ferrichrome receptor and the ferric iron ABC transporter are typically responsible for the transportation of siderophores, whereas the bacterioferritin and the bacterioferritin-associated ferredoxin are often responsible for iron storage and iron release under iron depleted conditions, respectively^35^. Upregulation of these genes responsible for iron acquisition suggest that initial competition for iron could contribute to selection of survivor *P. putida*. Iron competition is common in bacterial interactions^36–39^. Previously enhanced production of the primary myxobacterial siderophore myxochelin by *M. xanthus* contributed to an iron-restrictive environment and triggered production of the pigment actinorhodin from *S. coelicolor*^37,40^.

**Figure 7:**
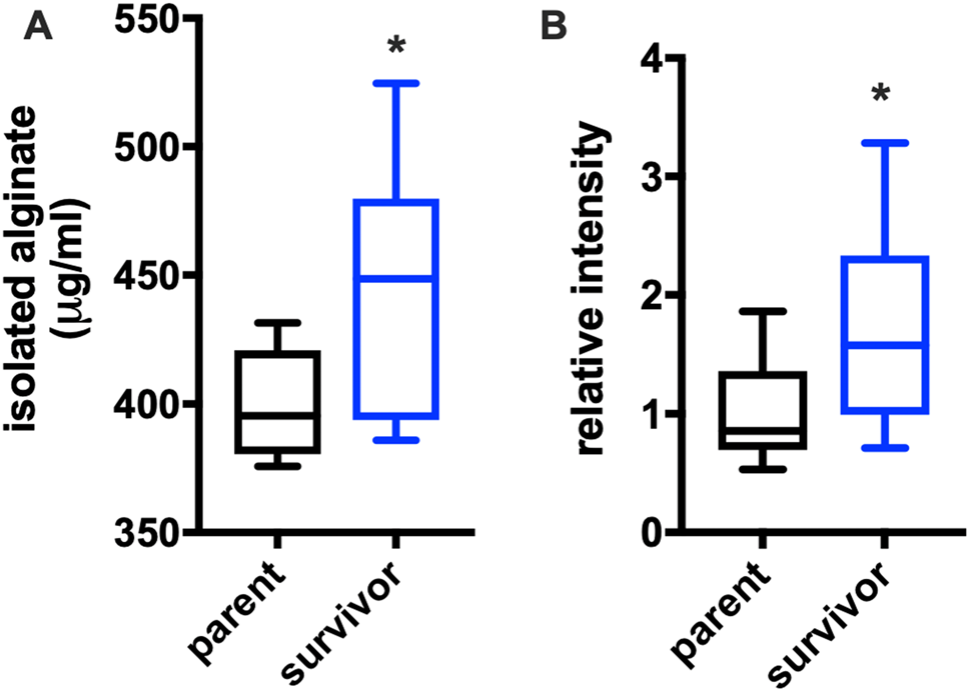
Increased alginate production from survivor *P. putida*. (**A**) Isolated alginate concentration determined from carbazole assays depicting increased alginate production observed from survivor *P. putida* (n = 16; *p* ≤ 0.02). (**B**) Relative intensities of alginate dot blots generated from isolated alginate also depicting increased alginate production observed from survivor *P. putida* (n = 10; *p* = 0.039). Relative units determined from average of control values with statistical significance calculated from an unpaired t test with Welch’s correction.

A four-gene operon including a ferric uptake regulatory (*furA*) associated gene (WP_016501343.1), a fumarate hydratase (WP_016501342.1), a manganese superoxide dismutase (*sodA*) (WP_016501340.1), and a hypothetical protein with a conserved DUF2753 domain (WP_06501341.1) was significantly up-regulated in survivor *P. putida* (Fig. 5B,C). Interestingly, expression of this operon from *P. aeruginosa* in response to iron limitation has been associated with elevated alginate production, an exopolysaccharide composed of mannuronic acid and guluronic acid monomers, and increased mucoidy^41,42^. This also corroborates our observation of small colony variance from survivor *P. putida*, as *P. aeruginosa* production of alginate overproduction is associated with small colony variants^41,42^. Notably, the alginate regulatory elements MucA (WP_016498171.1) and MucB (WP_016498172.1) were both significantly down regulated in survivor *P. putida* (Fig. 5B). The anti-sigma factor MucA and the associated binding partner MucB inhibit mucoid conversion by sequestering the alternative sigma factor AlgU required for alginate production in *P. aeruginosa*^43–46^. Increased transcription from the four-gene *furA* operon, which also belongs to the AlgU regulon, combined with decreased transcription of MucA and MucB, indicates that mucoid conversion might contribute to predator avoidance which parallels *Pseudomonas* sp. CM10, *P. aeruginosa,* and *S. marcescens* predator avoidance mechanisms when exposed to grazing amoeba^26–28,47,48^. An annotated alginate lyase present in *P. putida* (WP_016449106.1) was also down-regulated > 500-fold. This provides an additional explanation for survivor *P. putida* mucoidy since alginate lyases are responsible for the degradation of alginate^49^. Utilizing an established carbazole assay as well as alginate antibody dot blots^50–52^, we sought to determine if the increased transcription of genes associated with mucoid conversion observed from survivor *P. putida* resulted in increased production of alginate when compared to parent *P. putida*. Comparing supernatants, we observed that survivor *P. putida* indeed produced more alginate when compared to parent *P. putida* (Fig. 7). This result confirms increased production of alginate by survivor *P. putida* and provides an additional example of alginate overproduction in response to predatory stress by a *Pseudomonas* species.

**Figure 8:**
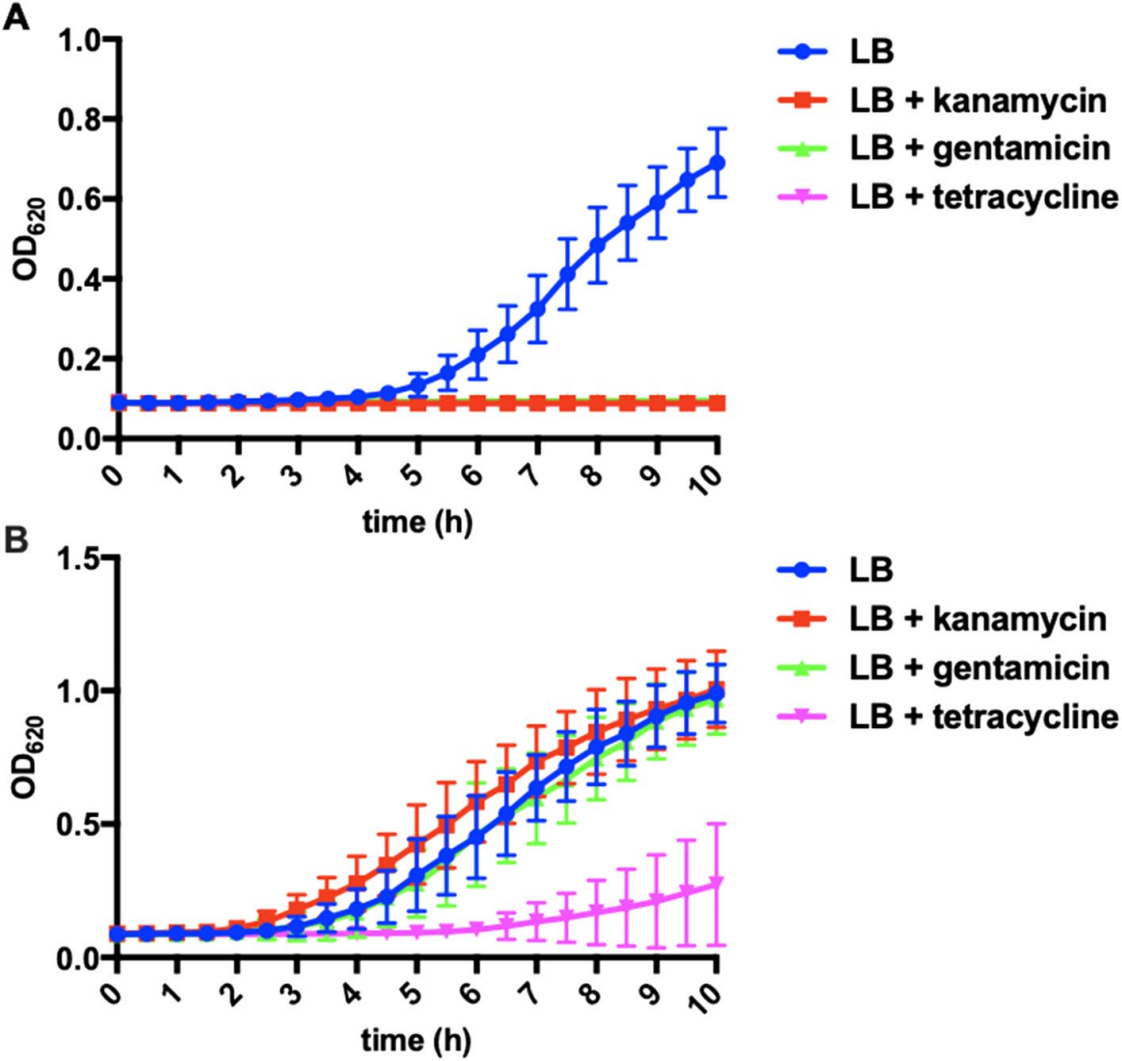
Antibiotic resistance of survivor *P. putida.* Growth curves for parent *P. putida* (**A**) and survivor *P. putida* (**B**) depicting antibiotic resistance unique to the survivor phenotype (n = 12). LB supplemented with gentamicin (10 μg/ml), kanamycin (50 μg/ml), and tetracycline (10 μg/ml) where indicated.

Increased transcription of numerous efflux and transport proteins were among the most up-regulated genes observed from survivor *P. putida* (Fig. 5D). Upregulated efflux genes included inner (WP_016498130 and WP_016499477.1) and outer (WP_016498128.1) membrane components highly homologous to the AcrAD-TolC-type multidrug resistance-nodulation-division (RND) family of efflux pumps and the Mex RND efflux pumps known to facilitate *P. aeruginosa* resistance to aminoglycosides and tetracyclines^53–55^. Genes encoding features homologous to multidrug efflux pumps contributing to virulence of human pathogens were also up-regulated in survivor *P. putida* including a P-type ATPase (WP_016502100) and an additional RND/MmpL (Mycobacterial membrane protein Large) protein (WP_016500594.1) (Fig. 5D). Provided the observation that numerous transport and efflux-associated proteins were overexpressed in survivor *P. putida* (Fig. 5D) including RND-type efflux pumps known to contribute to *P. aeruginosa* antibiotic resistances^53,56^, we were interested to determine if survivor *P. putida* exhibited antibiotic resistance. Survivor *P. putida* was capable of initially forming colonies on LB agar supplemented with the antibiotics gentamicin (10 μg/ml), kanamycin (50 μg/ml), and tetracycline (10 μg/ml), with no colonies from parent *P. putida* observed on identical medias. Subsequent growth curve assays comparing parent *P. putida* with survivor *P. putida* when grown in LB and LB supplemented with antibiotics (Fig. 8A,B) confirmed that survivor *P. putida* was uniquely resistant to gentamicin, kanamycin, and tetracycline. These results combined with the overexpression of efflux-associated proteins observed from survivor *P. putida* confirms that *C. ferrugineus* selection provides prey that benefit from antibiotic resistance and suggests efflux-mediated antibiotic resistance to be involved in *P. putida* response to predatory stress.

Interestingly, other highly expressed genes by survivor *P. putida* included a formaldehyde dehydrogenase (Fdh) (WP_016497631.1). A recent observation suggests formaldehyde secretion and subsequent higher expression of formaldehyde dehydrogenase to be a predation-resistance trait of *P. aeruginosa*^11^. Higher expression of Fdh by survivor *P. putida* suggests a common strategy to avoid predation might be employed by *Pseudomonas* genus. A variety of membrane-associated proteins were also overexpressed in survivor *P. putida* including an osmotically inducible protein OsmY (WP_0016501632.1), an outer membrane protein A (OmpA) family protein (WP_016501196.1), an FKBP-type peptidyl-prolyl cis–trans isomerase (WP_0165010671), an aquaporin (WP_016498616.1), and a DUF1282 domain-containing hypothetical protein from the Yip1 superfamily (WP_016499858.1) (Fig. 5A). Afforded these observed differences in gene expression, utilizing classical microbiological assays, we sought to determine if survivor *P. putida* demonstrated increased production of the siderophore pyoverdine, increased mucoidy, and antibiotic resistance.

### Increased production of phenazine-1-carboxylic acid by survivor P. putida

**Figure 9:**
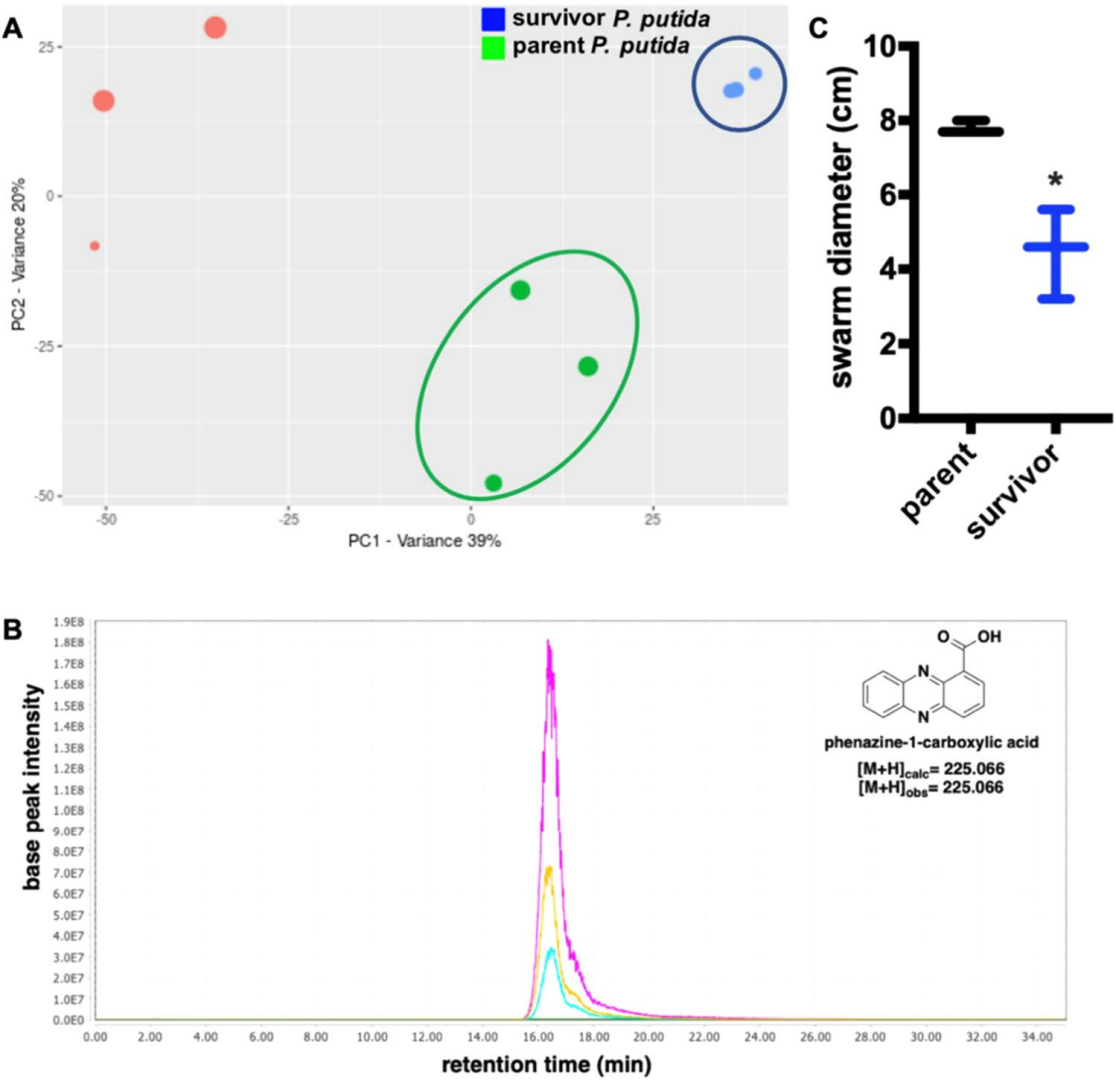
Phenazine-1-carboxylic acid production by survivor *P. putida* and potential contribution to predator avoidance trait. (**A**) Principal component analysis (PCA) score plot of untargeted mass spectrometry data from crude extracts of parent *P. putida* (green), survivor *P. putida* (blue), and media extracts with no bacteria added as a control (red). XCMS v3.7.1 was used to render the PCA score plot. (**B**) Extracted ion chromatograph (224.9–225.1 m/z) depicting presence of phenazine-1-carboxylic acid in extracts from survivor *P. putida* (n = 3; magenta, yellow, cyan) and absence in extracts from parent *P. putida* replicates (n = 3; red, blue, green (baseline)). Chromatograph rendered with MZmine v2.37. (**C**) Comparison of swarm diameters for *C. ferrugineus* cultivated on filter-sterilized supernatants from parent *P. putida* and survivor *P. putida* cultivation broths (n = 3; *p* = 0.039). Statistical significance calculated from an unpaired t test with Welch’s correction.

To identify differences in detectable quantities of *P. putida* metabolites, we utilized untargeted mass spectrometry to compare organic phase extracts from parent *P. putida* and survivor *P. putida*. Initial principle component score plot analysis of resulting data rendered by the metabolomic platform XCMS-MRM^57^ demonstrated survivor *P. putida* is metabolically distinct from parent *P. putida* (Fig. 9A). Additional analysis using Global Natural Product Social Molecular Networking (GNPS)^58^ resulted in a library hit for phenazine-1-carboxylic acid (PCA) ([M + H] = 225.066) that was only observed in extracts and supernatants from survivor *P. putida* (Fig. 9B and Supplemental Fig. 1). However, no significant change in expression of putative phenazine biosynthesis genes *phzD* (SUD74861.1) and *phzF* (SUD71919.1) was observed from survivor *P. putida* in our comparative transcriptomic data^59–62^. Interestingly, phenazine production has been observed to protect *Pseudomonas aureofaciens* from predatory myxobacteria^63^. To determine if PCA biosynthesis and observed differences in metabolic profiles contribute to predator avoidance, we conducted swarming assays with *C. ferrugineus* on media supplemented with filter-sterilized supernatants from parent *P. putida* and survivor *P. putida* cultivation broths. Swarming diameters of *C. ferrugineus* grown on medias with survivor *P. putida* supernatants were significantly smaller than the swarm diameters when grown on parent *P. putida* supernatants (Fig. 9C). These results suggest that survivor *P. putida* produces significantly greater quantities of PCA when compared to parent *P. putida*, and PCA production as well as observed differences in detected metabolic features may contribute to predator avoidance.

## Discussion

Compared to *P. putida* traits associated with protozoan predator avoidance^14^, resiliency of *P. putida* during bacterial predator–prey interactions remains underexplored. Predator–prey experiments that utilize the model myxobacterium *M. xanthus* with various prey such as *B. subtilis*^9,18,19^, *E. coli*^5,6^, *S. melilotti*^21,22^, *P. aeruginosa*^11^, and *S. coelicolor*^40^ have demonstrated that predatory stress selects for traits that benefit predator avoidance. Investigating the predator–prey interaction between *C. ferrugineus* and *P. putida*, we observed a *P. putida* phenotype that more capably avoids predation when compared to parent *P. putida*. Specifically, out of 100 independent predation assays conducted on both nutrient-free and nutrient-rich medias, we observed selection of a survivor *P. putida* phenotype 5 times. Observing this survivor phenotype on both nutrient-free and nutrient-rich medias, we concluded that selection of the phenotype was not dependent upon nutrient availability. Ultimately, we suggest that predatory stress selects for survivor *P. putida* and not other general stressors such as starvation.

Comparative transcriptomics provided up-regulated features that potentially contribute to the predator avoidance trait of survivor *P. putida*. Considering iron as an essential micronutrient for all life forms^36–39,64^, up-regulation of genes included in the biosynthetic pathway of the siderophore pyoverdine, increased fluorescence attributable to pyoverdine titers in extracellular extracts, and up-regulation of several features associated with iron acquisition and storage^35^ suggest a competitive environment for the acquisition of iron may contribute to survivor *P. putida* selection. Overall, these observations depict an iron-depleted predator–prey environment similar to the previously reported interaction between *M. xanthus* and *S. coelicolor*^37,40^*.* Despite no significant change in expression of putative phenazine biosynthesis genes *phzD* (SUD74861.1) and *phzF* (SUD71919.1) observed in our comparative transcriptomic data^59–62^, comparative metabolomics revealed detectible quantities of PCA in survivor *P. putida* extracts and not present in parent *P. putida* extracts. We suspect that the absence of significantly impacted transcription of genes involved in phenazine biosynthesis are the result of differences in survivor *P. putida* cultivation conditions during transcriptomic and metabolomic experiments and analysis of a single time point during our comparative transcriptomics. Interestingly, *P. aeruginosa* production of phenazines contributes to redox maintenance during biofilm formation and promotes efflux-based antibiotic resistance^56,65–67^, and we suspect that higher quantities of PCA detected in extracts from survivor *P. putida* may contribute to the increased transcription of efflux pump components. However, associations between phenazine production and efflux-mediated resistance have only been reported from *P. aeruginosa*, and any parallels potentially observed in our data require further investigation. Phenazine production has also previously been observed to protect *Pseudomonas aureofaciens* from predatory myxobacteria^63^, and provide *P. aeruginosa* a competitive advantage against *E. coli*^68^. Increased production of both pyoverdine and PCA indicate their biosynthesis to be a strategy commonly employed by pseudomonads to compete for the limited resources including competition during predator–prey interactions.

Several features associated with the AlgU regulon and mucoid conversion were also up-regulated in survivor *P. putida* including a four-gene operon associated with iron limitation conditions in *P. aeruginosa* and the regulatory transcription factors MucA and MucB were downregulated^41–43,45^. While the decreased transcription of regulatory MucA and MucB would suggest activation of genes within the AlgU regulon, these were the only significantly impacted features associated with the AlgU regulon in our transcriptomic analysis^69,70^. Again, we suspect that the absence of significantly impacted transcription of genes involved in alginate biosynthesis are the result of analyzing a single time point during our comparative transcriptomics. We do however observe a > 500-fold decrease in the transcription of an alginate lyase from survivor *P. putida* (WP_016449106.1) which could also contribute to the observed mucoidy. Considering the previously reported activation of the AlgU regulon in *P. putida* by membrane stress^71,72^, we would expect to see a significant response from transcriptomic analysis of survivor *P. putida* cells taken directly from predator–prey assays with *C. ferrugineus*. Other than this alginate lyase, down-regulated genes in survivor *P. putida* were abundant in numerous functional categories making it difficult to associate any of the observed phenotypic characteristics with specific features. The biological systems with the most down-regulated features included transcriptional regulators and signal transduction; replication, recombination, and repair; cell wall, membrane, and envelope biogenesis; and inorganic ion transport and metabolism.

We also observed an upregulated OmpA family protein in survivor *P. putida*. This OmpA family protein might also correlate with mucoid conversion in survivor *P. putida* as *P. aeruginosa* phenotypes that up-regulate expression of OmpA also overproduce alginate^73^. We confirmed increased alginate production by survivor *P. putida* using established carbazole assays and anti-alginate dot blots. Alginate-based mucoid conversion, microcolonies, and biofilm formation have previously been associated with *Pseudomonas* sp. CM10 and *P. aeruginosa* avoidance of grazing amoeba and could also contribute to the small colony variation observed from survivor *P. putida*^26,27,47^. Additionally, adaptive mucoid phenotypes have been previously observed in response to myxobacterial predation^5^, amoebal grazing^27^, macrophages^74^, and lytic phages^75^. These results and the noted difference in *C. ferrugineus* swarming during predator–prey interactions with survivor *P. putida* parallel previous observation of mucoid *S. meliloti* phenotypes that avoid *M. xanthus* predation^22^. These results provide clear overlap between features utilized by different *Pseudomonas* species under the predatory stress. The findings also afford additional evidence that the coincidental selection of prey virulence factors reported from amoebal predatory stress^12^ might also be observed from predator–prey interactions involving myxobacteria.

Comparative transcriptomics also revealed the up-regulated key features associated with efflux pumps. Of these, 3 putative RND family transporter components with high homology to the MexAB-OprM and MexCD-OprM efflux systems known to mediate aminoglycoside and tetracycline antibiotic resistance in *P. aeruginosa* were up-regulated in survivor *P. putida*^53,55^. Growth curve analysis determined that survivor *P. putida* was indeed resistant to the aminoglycosides kanamycin and gentamicin as well as tetracycline. Whereas, susceptibility to all 3 of these antibiotics was observed from parent *P. putida*. Interestingly, both efflux-mediated antibiotic resistance and mucoid conversion have also been attributed to virulence of clinical isolates of *P. aeruginosa*^44,76–78^.

It is worth noting that predatory stress contributes to the development of phenotypic traits that overlap traits observed from persistent clinical pathogens^12,16,76^. As an example, clinical isolates of persistent *P. aeruginosa* from cystic fibrosis patients exhibit increased mucoidy and small colony variation as well as efflux-mediated antibiotic resistance^78–80^. Other human pathogens that have demonstrated overlapping factors involved in both virulence and anti-predation strategies include *Legionella pneumophila*, *E. coli*, *Vibrio cholerae*, *Campylobacter jejuni*, *Listeria monocytogenes*, *Mycobacterium leprae*, and *Yersinia enterocolitica*^12,16^. Overlapping factors thus far observed to be associated with both virulence and predator avoidance include biofilm formation^15,26,48^, quorum signal^81^ and siderophore^82^ small molecule biosynthesis, toxin production^83–85^, expression of transport proteins such as type III and type VI secretion systems^86,87^ and antibiotic resistance-associated efflux pumps^88,89^. While the impact of predatory selection on virulence factors have primarily been reported from predator–prey experiments with grazing amoeba, efforts to determine the influence of generalist predators on microbial communities have suggested that bacterial predators also drive adaptation of prey traits that overlap with virulence in bacterial pathogens^5^. Recently, Nair et al*.* reported that during predator–prey interactions with *E. coli* prey, the predatory myxobacterium *M. xanthus* selects for 2 traits associated with virulence, mucoidy and increased expression of the outer membrane protease OmpT^5^. Our study adds to these findings and provides the importance of exploring bacterial predator–prey interactions to unravel features that parallel the emergence of clinical pathogens.

## Materials and methods

### Bacterial strains and cultivation

The myxobacterium *Cystobacter ferrugineus* strain Cbfe23, DSM 52764 was employed as the predator throughout. *Pseudomonas putida* type strain, ATCC 12633, and the discussed survivor *P. putida* resulted from predation assays with this parent *P. putida* type strain were included as prey. *C. ferrugineus* was grown on VY/2 solid (1.4% w/v agar, 0.1% w/v CaCl_2_ * 2 H_2_O, 0.5% w/v Baker’s yeast, 500 μM vitamin B12) media for 5–7 days. Luria–Bertani (LB) solid (1.5% agar) and liquid media were utilized for the cultivation of *P. putida*. Predation experiments were performed on WAT agar (1.5% w/v agar, 0.1% w/v CaCl_2_, 20 mM HEPES) plates. All bacteria were grown at 30 °C.

### Predation assays

Prey spot assay was performed as described by Seccareccia et al.^24^. Briefly, *P. putida* prey was grown in LB liquid media on a rotary shaker (150 rpm) at 30 °C for 16–18 h. Next, the bacterial cultures were sedimented by centrifugation at 4,000 g for 15 min, and the sedimented cells were washed and resuspended in TM buffer (50 mM Tris, pH 7.8, 10 mM MgSO_4_) to an OD_600_ of 0.5. Then the WAT agar plates were spotted with 150 µl of the freshly prepared bacterial suspensions, and the spots were dried in a laminar flow hood. Finally, *C. ferrugineus* was grown on VY/2 agar for 6 days before a 1 cm^2^ patch of cells was aseptically removed to inoculate the edge of the prey spot. The assay plates were incubated at 30 °C for 14 days or until the full visible swarming of *C. ferrugineus* on the prey spot. The assay was performed with 100 replicates. Any prey spot with visible biomass of *P. putida* after day 14 was considered as survivor *P. putida*, and five observed survivor *P. putida* stocks were acquired that remained visible after day 14 of the assay.

For subsequent predation assays, parent *P. putida* and survivor *P. putida* were included as prey. Predation assays were performed as mentioned above. Statistical significance of recorded time to predation reported in was calculated using an unpaired t test with Welch’s correction in Prism 7.0d. Subsequent predation assays were performed on VY/2 (1.4% w/v agar, 0.1% w/v CaCl_2_ * 2 H_2_O, 0.5% w/v Baker’s yeast, 500 μM vitamin B12) media as well, considered as nutrient-rich media. For distant spot predation assays, *C. ferrugineus* was inoculated at a distance of 5–7 mm from the established parent *P. putida* and survivor *P. putida* prey spots. The time (in days) *C. ferrugineus* took to contact prey and the time (in days) required for complete swarming of prey biomass were both recorded. Distant spot predation assays were performed with 8 replicates per phenotype. Statistical significance of recorded time to predation reported in days and recorded time to initial contact between *C. ferrugineus* and prey reported in days was calculated using an unpaired t test with Welch’s correction in Prism 7.0d. All predation assays were imaged daily using an AmScope SM-1TSW2-L6W-10M digital stereo microscope (AmScope, Irvine, CA).

### CFU assays and growth curve comparisons

The colony forming units (CFU) were calculated as described by DePas et al*.*^17^. Briefly, after the full swarming of *C. ferrugineus* over the prey spot, the agar slab under the prey spot was excised and suspended in 2 mL phosphate buffer saline (PBS). Next, a dilution series from this solution was inoculated on the LB plates, and the plates were incubated at 30 °C for 18 h. *Cystobacter ferrugineus* is unable to grow on LB media and was not observed during CFU assays. Then, CFU/mL was calculated from the viable colonies that appeared on the LB plates. Finally, the CFU/mL of survivor *P. putida* was compared with the CFU/mL of parent *P. putida*. Final biomass calculations and comparative growth curve data for parent and survivor *P. putida* were done by growing 8 replicates of each phenotype in 150 μl LB in well plates at 30 °C in an incubated plate reader (ThermoFisher) monitoring OD_620_ over 16 h with viable colonies quantified on LB plates after 16 h growth used to determine final biomass. Statistical significance of all CFU assays and doubling times were calculated using an unpaired t test with Welch’s correction and the exponential growth equation respectively in Prism 7.0d.

### RNA sequencing

Triplicate samples of parent *P. putida* and survivor *P. putida* grown from stocks in LB at 37 °C for 18 h and pelleted via centrifugation were stored in RNA*later* solution for sequencing. The DNA from parent *P. putida* (200 μl) was extracted using MagAttract HMW DNA Kit (Qiagen). The DNA was eluted in 100 uL AE buffer. The concentration of DNA was evaluated (Supplementary Table 1) using the Qubit dsDNA HS Assay Kit (Life Technologies). The library was prepared using Nextera DNA Flex library preparation kit (Illumina) following the manufacturer's user guide. An aliquot of 50 ng DNA was used to prepare the library. The sample underwent the simultaneous fragmentation and addition of adapter sequences. These adapters are utilized during a limited-cycle (6 cycles) PCR in which unique index was added to the sample. Following the library preparation, the final concentration of the library (Supplementary Table 1) was measured using the Qubit dsDNA HS Assay Kit (Life Technologies), and the average library size (Supplementary Table 1) was determined using the Agilent 2100 Bioanalyzer (Agilent Technologies). The library was diluted (to 6.5 pM) and sequenced paired end for 500 cycles using the MiSeq system (Illumina).

Total RNA was isolated from triplicate samples of parent *P. putida* and survivor *P. putida* using the RNeasy PowerSoil Total RNA Kit (Qiagen) following the manufacturer's instructions. A 400 μl cell sample was used for extractions. The concentration of total RNA was determined (Supplementary Table 2) using the Qubit RNA Assay Kit (Life Technologies). For rRNA depletion, first, 1000 ng of total RNA was used to remove the DNA contamination using Baseline-ZERO DNase (Epicentre) following the manufacturer's instructions followed by purification using the RNA Clean & Concentrator-5 columns (Zymo Research). DNA free RNA samples were used for rRNA removal by using RiboMinus rRNA Removal Kit (Bacteria; Thermo Fisher Scientific) and final purification was performed using the RNA Clean & Concentrator-5 columns (Zymo Research). rRNA depleted samples were used for library preparation using the KAPA mRNA HyperPrep Kits (Roche) by following the manufacturer's instructions. Following the library preparation, the final concentrations of all libraries (Supplementary Table 2) were measured using the Qubit dsDNA HS Assay Kit (Life Technologies), and the average library size was determined using the Agilent 2100 Bioanalyzer (Agilent Technologies). The libraries were then pooled in equimolar ratios of 0.6 nM, and sequenced paired end for 300 cycles using the NovaSeq 6000 system (Illumina). Differential expression between the resulting transcriptomes was calculated from pair-wise analysis of trimmed mean of M-values (TMM) normalized read counts^90^ including fold change, counts-per-million (CPM), and associated *p*-values using the R-package, edgeR^91^. Differential expression data with *p*-values ≤ 0.05 were considered statistically significant. Genome and RNA sequencing was conducted by MR DNA (Molecular Research LP). All fold change, CPM, and associated *p*-values resulting from differential expression experiments as described included as supplementary data file Supplemental Dataset 1 (survivor *P. putida* compared with parent *P. putida*). All raw RNAseq data is publicly available at the NCBI Sequence Read Archive (PRJNA577468).

### Assessment of extracellular pyoverdine

Extracellular pyoverdine fluorescence was detected from supernatants following methodologies Imperi et al*.* and Barrientos-Moreno et al*.*^33,34^. Clarified supernatants from overnight cultivation of 200 μl LB cultures of parent *P. putida* and survivor *P. putida* adjusted to an OD_600_ of 0.07 were generated by centrifugation (10,000 rpm, 15 m). Fluorescence was recorded at 455 nm upon excitation at 400 nm using a CLARIOstar microplate reader (BMG Labtech Inc., Cary, NC, USA). Sterile LB was used as a negative control to assess background fluorescence. Average relative fluorescence for supernatants from parent *P. putida* and survivor *P. putida* cultures (n = 12) are reported with statistical significance was calculated using an unpaired t test with Welch’s correction in Prism 7.0d.

### Metabolite extraction and analysis

For metabolomic analysis, survivor *P. putida* and parent *P. putida* were cultivated on VY/2 agar plates as described in the predation assay. VY/2 agar plates with TM buffer were used as a negative control. The plates were incubated at 30 °C for 14 days. After the incubation period, agar was chopped and extracted with ethyl acetate (EtOAC). The EtOAc extracts were dried in vacuo to produce crude extracts for LC–MS/MS analysis. The crude extracts from each condition were generated in triplicate and analyzed as previously described^92^. LC–MS/MS generated data was converted to.mzML files using MS-Convert, and the GNPS platform^58^ was utilized as a dereplication tool to look for the library hits within publicly available natural products libraries. For the statistical analysis, using XCMSonline^57^, multigroup analysis with HPLC orbitrap default settings was employed. MZmine 2.53 was used to generate extracted ion chromatograms^93–95^.

### C. ferrugineus swarming assays with supernatants of parent P. putida and survivor P. putida culture broth

Parent *P. putida* and survivor *P. putida* were grown in LB media overnight to an OD_600_ of 1.5. Media supernatants were acquired after centrifuging the cultures at 10,000 rpm for 15 min. The supernatants were filter sterilized with a 0.2 µl filter. Aliquots of 1 mL for each filtered supernatant was used to make a uniform lawn on a VY/2 agar plate. *C. ferrugineus* was spotted in the middle of the lawn and incubated for 5 days at 30 °C. On the fifth day, the diameter of *C. ferrugineus* was measured.

### Carbazole assays

The alginate was isolated according to Jones et al.^96^. In brief, both parent *P. putida* and survivor *P. putida* were grown overnight in 2 ml LB media and adjusted to an OD_600_ of 1.00. Following centrifugation, 1 ml of supernatant was treated with 2% cetyl pyridinium chloride to precipitate the alginate. The precipitated alginate was further collected by centrifugation. It was resuspended in 1 M NaCl and then re-precipitated in cold isopropanol. Precipitated alginate was suspended in 150 μl 0.9% (w/v) saline solution. Following established protocols^50,51^, a 50 µL aliquot of the isolated alginate and a dilution series of standard alginic acid (Sigma) were mixed with 200 µL of a solution of 25 mM sodium tetraborate in sulfuric acid and added in a 96-well plate. Next, the plate was heated for 10 min at 100 °C in an oven. After cooling at room temperature for 15 min, 50 μl of a 0.125% carbazole solution in absolute ethanol was added. Then, the plate was re-heated at 100 °C for 10 min in an oven and cooled down at room temperature for 15 min. Finally, the plate was read in a CLARIOstar microplate reader (BMG Labtech Inc., Cary, NC, USA) at a wavelength of 550 nm. Quantities of alginate per sample (μg/ml) were calculated from the resulting standard curve of purchase alginic acid, and statistical significance was calculated using an unpaired t test with Welch’s correction in Prism 7.0d.

### Anti-alginate dot plots

Alginate dot blots were generated using methodology from Lorenz et al*.* with slight modifications^52^. Alginate was isolated as previously described, suspended in 150 μl 0.9% (w/v) saline solution, and 2 μl aliquots were pipetted onto a nitrocellulose membrane. After air-drying for 30 min, Tris buffered saline-Tween 20 (TBST) with 5% bovine serum albumin (BSA) was used for membrane blocking for 1 h. Membrane was then washed 3 × for 10 min with TBST and subsequently incubated with the alginate antibody (Sigma, monoclonal, anti-mouse) overnight at 4 °C. The following day, the membrane was washed 3 × for 10 min with TBST, incubated with IRDye 800CW Goat anti-mouse (Sigma) for 30 min, rinsed 2 × with TBST, and imaged on a LI-COR Odyssey. Average relative intensities for imaged blots comparing isolated alginate from parent *P. putida* with alginate isolated from survivor *P. putida* (n = 10) are reported with statistical significance was calculated using an unpaired t test with Welch’s correction in Prism 7.0d.

### Antibiotic susceptibility assays

The standard growth curve assay was employed to determine the sensitivity of parent *P. putida* and survivor *P. putida* towards kanamycin (50 mg/mL), gentamicin (10 mg/mL), and tetracycline (10 mg/mL) antibiotics. The Lauria Bertani (LB) broth without any supplemented antibiotics was used as a control. The test was performed in a 96-well microtiter plate. In a 96-well plate, each well including 200 µL of LB or LB supplemented with an antibiotic was inoculated with a colony of *P. putida.* Next, the absorbance at OD_620_ was recorded using a plate reader every 30 min for 10 h to examine the growth dynamics of each phenotype. The test was performed in 12 replicates per condition for both parent *P. putida* and survivor *P. putida*. Recorded OD_620_ values were plotted using Prism version 7.0d with error bars depicting standard deviation of replicate data.

## Supplementary Information

Supplementary Information 1.

Supplementary Information 2.

## References

[1] Albataineh H, Stevens DC (2018). Marine myxobacteria: A few good halophiles. Mar. Drugs.

[2] Findlay BL (2016). The chemical ecology of predatory soil bacteria. ACS Chem. Biol..

[3] Munoz-Dorado J, Marcos-Torres FJ, Garcia-Bravo E, Moraleda-Munoz A, Perez J (2016). Myxobacteria: Moving, killing, feeding, and surviving together. Front Microbiol..

[4] Mohr KI (2018). Diversity of myxobacteria-we only see the tip of the iceberg. Microorganisms.

[5] Nair RR (2019). Bacterial predator-prey coevolution accelerates genome evolution and selects on virulence-associated prey defences. Nat. Commun..

[6] Perez J, Moraleda-Munoz A, Marcos-Torres FJ, Munoz-Dorado J (2016). Bacterial predation: 75 years and counting!. Environ. Microbiol..

[7] Livingstone PG, Millard AD, Swain MT, Whitworth DE (2018). Transcriptional changes when Myxococcus xanthus preys on Escherichia coli suggest myxobacterial predators are constitutively toxic but regulate their feeding. Microb. Genom..

[8] Ellis BM, Fischer CN, Martin LB, Bachmann BO, McLean JA (2019). Spatiochemically profiling microbial interactions with membrane scaffolded desorption electrospray ionization-ion mobility-imaging mass spectrometry and unsupervised segmentation. Anal. Chem..

[9] Muller S (2016). Identification of functions affecting predator-prey interactions between Myxococcus xanthus and Bacillus subtilis. J. Bacteriol..

[10] Xiao Y, Wei X, Ebright R, Wall D (2011). Antibiotic production by myxobacteria plays a role in predation. J. Bacteriol..

[11] Sutton D, Livingstone PG, Furness E, Swain MT, Whitworth DE (2019). Genome-wide identification of myxobacterial predation genes and demonstration of formaldehyde secretion as a potentially predation-resistant trait of pseudomonas aeruginosa. Front Microbiol..

[12] Erken M, Lutz C, McDougald D (2013). The rise of pathogens: Predation as a factor driving the evolution of human pathogens in the environment. Microb. Ecol..

[13] Justice SS, Hunstad DA, Cegelski L, Hultgren SJ (2008). Morphological plasticity as a bacterial survival strategy. Nat. Rev. Microbiol..

[14] Seiler C (2017). Grazing resistance of bacterial biofilms: A matter of predators' feeding trait. FEMS Microbiol. Ecol..

[15] Weitere M, Bergfeld T, Rice SA, Matz C, Kjelleberg S (2005). Grazing resistance of Pseudomonas aeruginosa biofilms depends on type of protective mechanism, developmental stage and protozoan feeding mode. Environ. Microbiol..

[16] Sun S, Noorian P, McDougald D (2018). Dual role of mechanisms involved in resistance to predation by protozoa and virulence to humans. Front Microbiol..

[17] DePas WH (2014). Biofilm formation protects Escherichia coli against killing by Caenorhabditis elegans and Myxococcus xanthus. Appl. Environ. Microbiol..

[18] Muller S (2014). Bacillaene and sporulation protect Bacillus subtilis from predation by Myxococcus xanthus. Appl. Environ. Microbiol..

[19] Muller S, Strack SN, Ryan SE, Kearns DB, Kirby JR (2015). Predation by Myxococcus xanthus induces Bacillus subtilis to form spore-filled megastructures. Appl. Environ. Microbiol..

[20] Wang C (2019). Bacillus licheniformis escapes from Myxococcus xanthus predation by deactivating myxovirescin A through enzymatic glucosylation. Environ. Microbiol..

[21] Contreras-Moreno FJ (2020). Copper and melanin play a role in Myxococcus xanthus predation on Sinorhizobium meliloti. Front Microbiol..

[22] Perez J (2014). Rhizobial galactoglucan determines the predatory pattern of Myxococcus xanthus and protects Sinorhizobium meliloti from predation. Environ. Microbiol..

[23] Livingstone PG, Morphew RM, Whitworth DE (2017). Myxobacteria are able to prey broadly upon clinically-relevant pathogens, exhibiting a prey range which cannot be explained by phylogeny. Front Microbiol..

[24] Seccareccia I, Kost C, Nett M (2015). Quantitative analysis of lysobacter predation. Appl. Environ. Microbiol..

[25] Akbar S, Dowd SE, Stevens DC (2017). Draft Genome sequence of cystobacter ferrugineus strain Cbfe23. Genome Announc..

[26] Matz C, Bergfeld T, Rice SA, Kjelleberg S (2004). Microcolonies, quorum sensing and cytotoxicity determine the survival of Pseudomonas aeruginosa biofilms exposed to protozoan grazing. Environ. Microbiol..

[27] Matz C, Deines P, Jurgens K (2002). Phenotypic variation in Pseudomonas sp. CM10 determines microcolony formation and survival under protozoan grazing. FEMS Microbiol. Ecol..

[28] Queck SY, Weitere M, Moreno AM, Rice SA, Kjelleberg S (2006). The role of quorum sensing mediated developmental traits in the resistance of Serratia marcescens biofilms against protozoan grazing. Environ. Microbiol..

[29] Drake EJ (2007). The 1.8 A crystal structure of PA2412, an MbtH-like protein from the pyoverdine cluster of Pseudomonas aeruginosa. J. Biol. Chem..

[30] Felnagle EA (2010). MbtH-like proteins as integral components of bacterial nonribosomal peptide synthetases. Biochemistry.

[31] Parker DL (2014). Pyoverdine synthesis by the Mn(II)-oxidizing bacterium Pseudomonas putida GB-1. Front Microbiol..

[32] Ringel MT, Bruser T (2018). The biosynthesis of pyoverdines. Microb. Cell.

[33] Barrientos-Moreno L, Molina-Henares MA, Pastor-Garcia M, Ramos-Gonzalez MI, Espinosa-Urgel M (2019). Arginine biosynthesis modulates pyoverdine production and release in pseudomonas putida as part of the mechanism of adaptation to oxidative stress. J. Bacteriol..

[34] Imperi F, Tiburzi F, Visca P (2009). Molecular basis of pyoverdine siderophore recycling in Pseudomonas aeruginosa. Proc. Natl. Acad. Sci. U S A.

[35] Wandersman C, Delepelaire P (2004). Bacterial iron sources: From siderophores to hemophores. Annu. Rev. Microbiol..

[36] Harrison F, Paul J, Massey RC, Buckling A (2008). Interspecific competition and siderophore-mediated cooperation in Pseudomonas aeruginosa. ISME J..

[37] Lee N (2020). Iron competition triggers antibiotic biosynthesis in Streptomyces coelicolor during coculture with Myxococcus xanthus. ISME J..

[38] Traxler MF, Seyedsayamdost MR, Clardy J, Kolter R (2012). Interspecies modulation of bacterial development through iron competition and siderophore piracy. Mol. Microbiol..

[39] Weaver VB, Kolter R (2004). Burkholderia spp. alter Pseudomonas aeruginosa physiology through iron sequestration. J. Bacteriol..

[40] Perez J (2011). Myxococcus xanthus induces actinorhodin overproduction and aerial mycelium formation by Streptomyces coelicolor. Microb. Biotechnol..

[41] Hassett DJ (1997). An operon containing fumC and sodA encoding fumarase C and manganese superoxide dismutase is controlled by the ferric uptake regulator in Pseudomonas aeruginosa: Fur mutants produce elevated alginate levels. J. Bacteriol..

[42] Hassett DJ, Howell ML, Sokol PA, Vasil ML, Dean GE (1997). Fumarase C activity is elevated in response to iron deprivation and in mucoid, alginate-producing Pseudomonas aeruginosa: Cloning and characterization of fumC and purification of native fumC. J. Bacteriol..

[43] Damron FH, Goldberg JB (2012). Proteolytic regulation of alginate overproduction in Pseudomonas aeruginosa. Mol. Microbiol..

[44] Deretic V (1993). Conversion to mucoidy in Pseudomonas aeruginosa. Biotechnology (N. Y).

[45] Li S (2019). Structural basis for the recognition of MucA by MucB and AlgU in Pseudomonas aeruginosa. FEBS J..

[46] Martin DW, Schurr MJ, Mudd MH, Deretic V (1993). Differentiation of Pseudomonas aeruginosa into the alginate-producing form: Inactivation of mucB causes conversion to mucoidy. Mol. Microbiol..

[47] Matz C, Kjelleberg S (2005). Off the hook–how bacteria survive protozoan grazing. Trends Microbiol..

[48] Matz C (2005). Biofilm formation and phenotypic variation enhance predation-driven persistence of Vibrio cholerae. Proc. Natl. Acad. Sci. U S A.

[49] Boyd A, Chakrabarty AM (1994). Role of alginate lyase in cell detachment of Pseudomonas aeruginosa. Appl. Environ. Microbiol..

[50] Al Ahmar R, Kirby BD, Yu HD (2020). Culture of small colony variant of pseudomonas aeruginosa and quantitation of its alginate. J. Vis. Exp..

[51] Knutson CA, Jeanes A (1968). A new modification of the carbazole analysis: Application to heteropolysaccharides. Anal. Biochem..

[52] Lorenz C, Dougherty TJ, Lory S (2020). Transcriptional responses of pseudomonas aeruginosa to inhibition of lipoprotein transport by a small molecule inhibitor. J. Bacteriol..

[53] Aeschlimann JR (2003). The role of multidrug efflux pumps in the antibiotic resistance of Pseudomonas aeruginosa and other gram-negative bacteria: Insights from the Society of Infectious Diseases Pharmacists. Pharmacotherapy.

[54] Garneau-Tsodikova S, Labby KJ (2016). Mechanisms of resistance to aminoglycoside antibiotics: Overview and perspectives. Medchemcomm.

[55] Li XZ, Plesiat P, Nikaido H (2015). The challenge of efflux-mediated antibiotic resistance in Gram-negative bacteria. Clin. Microbiol. Rev..

[56] Sakhtah H (2016). The Pseudomonas aeruginosa efflux pump MexGHI-OpmD transports a natural phenazine that controls gene expression and biofilm development. Proc. Natl. Acad. Sci. U S A.

[57] Domingo-Almenara X (2018). XCMS-MRM and METLIN-MRM: A cloud library and public resource for targeted analysis of small molecules. Nat. Methods.

[58] Wang M (2016). Sharing and community curation of mass spectrometry data with Global Natural Products Social Molecular Networking. Nat. Biotechnol..

[59] Blankenfeldt W (2004). Structure and function of the phenazine biosynthetic protein PhzF from Pseudomonas fluorescens. Proc. Natl. Acad. Sci. U S A.

[60] Blankenfeldt W, Parsons JF (2014). The structural biology of phenazine biosynthesis. Curr. Opin. Struct. Biol..

[61] Mavrodi DV (2010). Diversity and evolution of the phenazine biosynthesis pathway. Appl. Environ. Microbiol..

[62] Parsons JF (2004). Structure and function of the phenazine biosynthesis protein PhzF from Pseudomonas fluorescens 2–79. Biochemistry.

[63] Bull CT, Shetty KG, Subbarao KV (2002). Interactions between myxobacteria, plant pathogenic fungi, and biocontrol agents. Plant. Dis..

[64] Andrews SC, Robinson AK, Rodriguez-Quinones F (2003). Bacterial iron homeostasis. FEMS Microbiol. Rev..

[65] Dietrich LE, Price-Whelan A, Petersen A, Whiteley M, Newman DK (2006). The phenazine pyocyanin is a terminal signalling factor in the quorum sensing network of Pseudomonas aeruginosa. Mol. Microbiol..

[66] Schiessl KT (2019). Phenazine production promotes antibiotic tolerance and metabolic heterogeneity in Pseudomonas aeruginosa biofilms. Nat. Commun..

[67] Sporer AJ (2018). Pseudomonas aeruginosa PumA acts on an endogenous phenazine to promote self-resistance. Microbiology (Reading).

[68] Khare A, Tavazoie S (2015). Multifactorial Competition and resistance in a two-species bacterial system. PLoS Genet..

[69] Markel E, Stodghill P, Bao Z, Myers CR, Swingle B (2016). AlgU controls expression of virulence genes in pseudomonas syringae pv. tomato DC3000. J. Bacteriol..

[70] Wood LF, Ohman DE (2009). Use of cell wall stress to characterize sigma 22 (AlgT/U) activation by regulated proteolysis and its regulon in Pseudomonas aeruginosa. Mol. Microbiol..

[71] Ainsaar K, Tamman H, Kasvandik S, Tenson T, Horak R (2019). The TonBm-PocAB system is required for maintenance of membrane integrity and polar position of flagella in pseudomonas putida. J. Bacteriol..

[72] Mumm K, Ainsaar K, Kasvandik S, Tenson T, Horak R (2016). Responses of pseudomonas putida to zinc excess determined at the proteome level: Pathways dependent and independent of ColRS. J. Proteome Res..

[73] Yang F (2018). PA0833 is an OmpA C-like protein that confers protection against pseudomonas aeruginosa infection. Front Microbiol..

[74] Miskinyte M (2013). The genetic basis of Escherichia coli pathoadaptation to macrophages. PLoS Pathog..

[75] Scanlan PD, Buckling A (2012). Co-evolution with lytic phage selects for the mucoid phenotype of Pseudomonas fluorescens SBW25. ISME J..

[76] Leong W, Lutz C, Williams J, Yan HP, Benny YKY, Chua C, Rice SA, Givskov M, Sanderson-Smith M, McDougald D (2020). *Pseudomonas aeruginosa* isolates co-incubated with *Acanthamoeba castellanii* exhibit phenotypes similar to chronic cystic fibrosis isolates. bioRxiv.

[77] Mulcahy LR, Burns JL, Lory S, Lewis K (2010). Emergence of Pseudomonas aeruginosa strains producing high levels of persister cells in patients with cystic fibrosis. J. Bacteriol..

[78] Winstanley C, O'Brien S, Brockhurst MA (2016). Pseudomonas aeruginosa evolutionary adaptation and diversification in cystic fibrosis chronic lung infections. Trends Microbiol..

[79] Malone JG (2015). Role of small colony variants in persistence of Pseudomonas aeruginosa infections in cystic fibrosis lungs. Infect. Drug Resist..

[80] Maunders E, Welch M (2017). Matrix exopolysaccharides; the sticky side of biofilm formation. FEMS Microbiol. Lett..

[81] Sun S, Kjelleberg S, McDougald D (2013). Relative contributions of Vibrio polysaccharide and quorum sensing to the resistance of Vibrio cholerae to predation by heterotrophic protists. PLoS ONE.

[82] Butt AT, Thomas MS (2017). Iron acquisition mechanisms and their role in the virulence of burkholderia species. Front Cell. Infect. Microbiol..

[83] Chekabab SM, Daigle F, Charette SJ, Dozois CM, Harel J (2013). Shiga toxins decrease enterohaemorrhagic Escherichia coli survival within Acanthamoeba castellanii. FEMS Microbiol. Lett..

[84] Lainhart W, Stolfa G, Koudelka GB (2009). Shiga toxin as a bacterial defense against a eukaryotic predator, Tetrahymena thermophila. J. Bacteriol..

[85] Steinberg KM, Levin BR (2007). Grazing protozoa and the evolution of the Escherichia coli O157:H7 Shiga toxin-encoding prophage. Proc. Biol. Sci..

[86] Matz C (2008). Pseudomonas aeruginosa uses type III secretion system to kill biofilm-associated amoebae. ISME J..

[87] Riquelme S (2016). Relevant genes linked to virulence are required for salmonella typhimurium to survive intracellularly in the social amoeba dictyostelium discoideum. Front Microbiol..

[88] Pombinho R (2017). Listeria monocytogenes CadC regulates cadmium efflux and fine-tunes lipoprotein localization to escape the host immune response and promote infection. J. Infect. Dis..

[89] Vieira A, Ramesh A, Seddon AM, Karlyshev AV (2017). CmeABC multidrug efflux pump contributes to antibiotic resistance and promotes campylobacter jejuni survival and multiplication in acanthamoeba polyphaga. Appl. Environ. Microbiol..

[90] Dillies MA (2013). A comprehensive evaluation of normalization methods for Illumina high-throughput RNA sequencing data analysis. Brief Bioinform..

[91] Robinson MD, McCarthy DJ, Smyth GK (2010). edgeR: A Bioconductor package for differential expression analysis of digital gene expression data. Bioinformatics.

[92] Adaikpoh BI (2020). Myxobacterial response to methyljasmonate exposure indicates contribution to plant recruitment of micropredators. Front Microbiol..

[93] Katajamaa M, Miettinen J, Oresic M (2006). MZmine: Toolbox for processing and visualization of mass spectrometry based molecular profile data. Bioinformatics.

[94] Olivon F, Grelier G, Roussi F, Litaudon M, Touboul D (2017). MZmine 2 data-preprocessing to enhance molecular networking reliability. Anal. Chem..

[95] Pluskal T, Castillo S, Villar-Briones A, Oresic M (2010). MZmine 2: Modular framework for processing, visualizing, and analyzing mass spectrometry-based molecular profile data. BMC Bioinform..

[96] Jones CJ, Ryder CR, Mann EE, Wozniak DJ (2013). AmrZ modulates Pseudomonas aeruginosa biofilm architecture by directly repressing transcription of the psl operon. J. Bacteriol..

