# Supplementary material for: Functional genomics study of Pseudomonas putida to determine traits associated with avoidance of a myxobacterial predator
Source: Sci Rep. 2021 Aug 12;11:16445. doi: 10.1038/s41598-021-96046-8 (PMC8360965; doi:10.1038/s41598-021-96046-8)
Supplement: Supplementary file 1 — Supplementary Information 1. [file 41598_2021_96046_MOESM1_ESM.docx]

**Predatory selection and multiomics analysis of *Pseudomonas putida* phenotype to determine traits associated with avoidance of myxobacterial predator**

Shukria Akbar^a^ and D. Cole Stevens^a,#^

Department of BioMolecular Sciences, University of Mississippi, University, MS, USA^a^

#Address correspondence to D. Cole Stevens, stevens@olemiss.edu.

**Supplementary Material**

| **Sample ID** | **DNA concentration (ng/µL)** | **Final library DNA concentration (ng/µL)** | **Average Library**  **size (bp)** |
| --- | --- | --- | --- |
| PputidaTypestrain-3DNA | 34.0 | 18.20 | 901 |

**Supplementary Table 1:** Concentration of total DNA, final library concentration, and average library size for *P. putida* type strain sample used for genome sequencing.

| Sample | RNA Concentration (ng/uL) | Library Concentration (ng/uL) | Avg Library size (bp) |
| --- | --- | --- | --- |
| PputidaTypestrain-1 | 2360.0 | 39.60 | 610 |
| PputidaTypestrain-2 | 2360.0 | 37.60 | 591 |
| PputidaTypestrain-3 | 2360.0 | 43.80 | 634 |
| SurvivorPputida-1 | 2280.0 | 44.00 | 574 |
| SurvivorPputida-2 | 2560.0 | 32.60 | 435 |
| SurvivorPputida-3 | 1728.0 | 43.00 | 523 |

**Supplementary Table 2:** Concentration of total RNA, final library concentration, and average library size for *P. putida* type strain (predator unexposed control) and *P. putida* survivor phenotype.


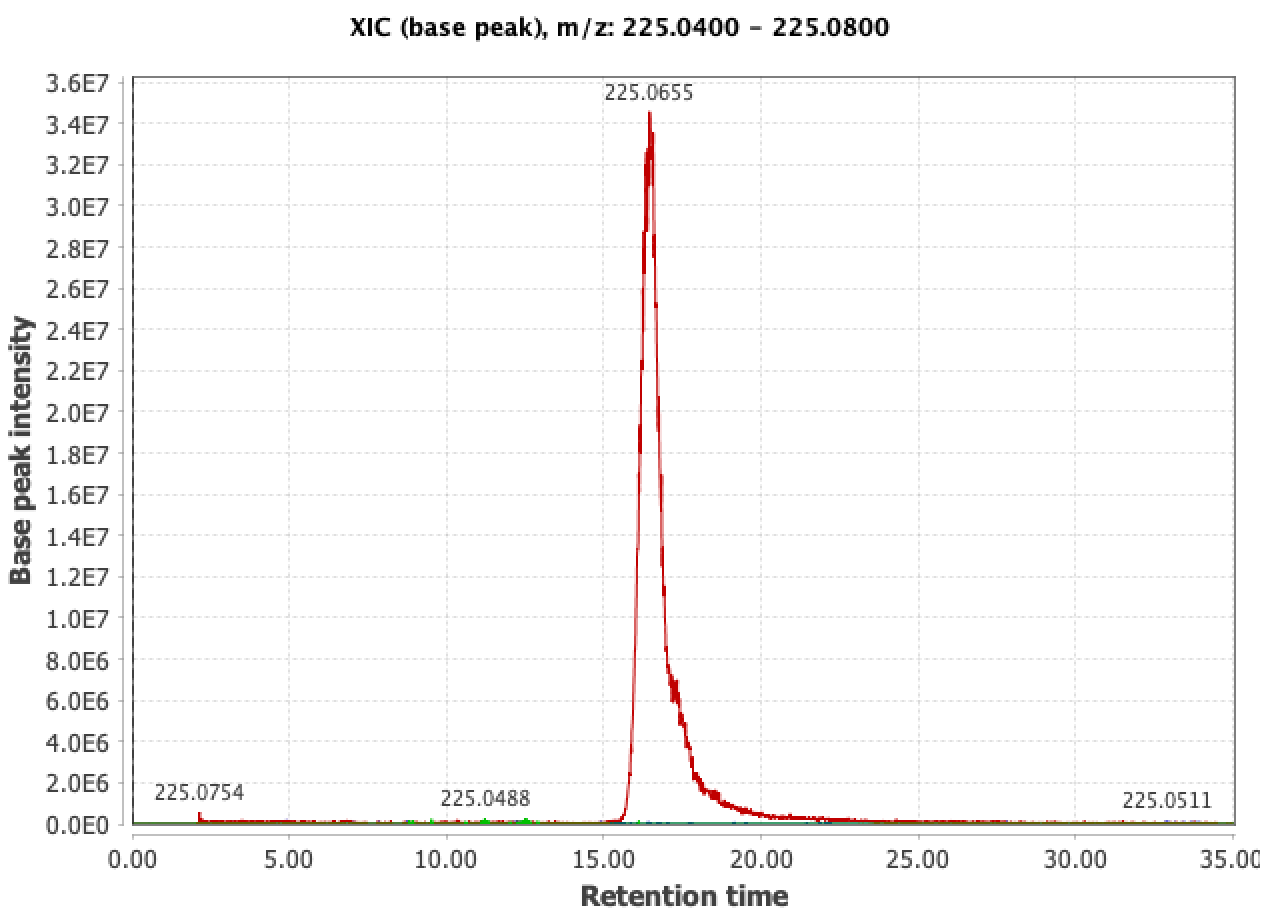
**Supplemental Figure 1:** Extracted ion chromatograph (225.04-225.08 m/z) depicting presence of phenazine-1-carboxylic acid ([M+H]_calc_=225.066) in media supernatants from survivor *P. putida* (red) and absence in extracts from parent *P. putida* (blue baseline) and media control (green baseline).


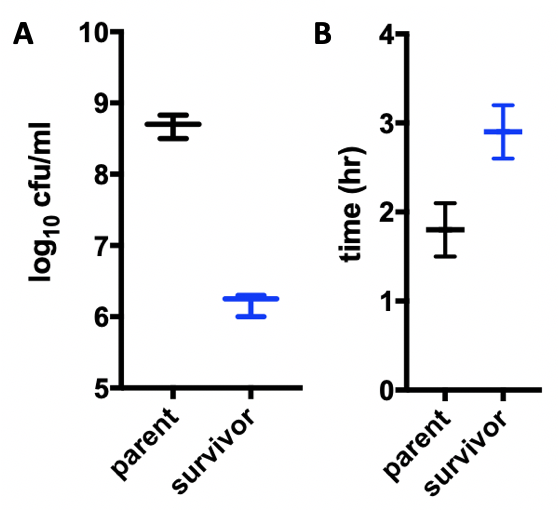


**Supplemental Figure 2:** A) Biomass comparison of parent and survivor *P. putida* after 16 hr growth in LB at 30 °C reported in log_10_ CFUs/ml. B) Doubling time comparison comparison of parent and survivor *P. putida*.
